# Association of rheumatoid arthritis with bronchial asthma and asthma-related comorbidities: A population-based national surveillance study

**DOI:** 10.3389/fmed.2023.1006290

**Published:** 2023-03-10

**Authors:** Jung Gon Kim, Jiyeon Kang, Joo-Hyun Lee, Hyeon-Kyoung Koo

**Affiliations:** ^1^Division of Rheumatology, Department of Internal Medicine, Ilsan Paik Hospital, Inje University College of Medicine, Goyang-si, Republic of Korea; ^2^Division of Pulmonary and Critical Care Medicine, Department of Internal Medicine, Ilsan Paik Hospital, Inje University College of Medicine, Goyang-si, Republic of Korea

**Keywords:** asthma, rheumatoid arthritis, obesity, sinusitis, allergy

## Abstract

**Background:**

The aim of this study was to investigate the impact of rheumatoid arthritis (RA) on the prevalence of bronchial asthma and asthma-related comorbidities. We also aimed to identify the influence of RA on interrelationship between asthma and asthma-related comorbidities.

**Methods:**

From the Korean National Health and Nutrition Examination Survey, participants >40 years of age who completed questionnaires and spirometry tests were enrolled. Patient data on RA, asthma, allergic rhinitis, atopic dermatitis, chronic obstructive pulmonary disease (COPD), sinusitis, otitis media, and body mass index (BMI) were collected. Logistic regression and network analyses were performed.

**Results:**

A total of 14,272 subjects were enrolled, among which, 334 (2.4%) had RA. RA was significantly associated with asthma (OR 2.32; 95% CI 1.51–3.57), allergic rhinitis (OR 1.51; 95% CI 1.08–2.10), and sinusitis (OR 1.64; 95% CI 1.08–2.50). The network analysis of total patients revealed a positive interrelationship between asthma and allergic rhinitis, sinusitis, otitis media, atopic dermatitis, BMI, and RA. The interrelationship between asthma and sinusitis was stronger in the RA group. Of note, the relationship between asthma and BMI was distinctively found only in the RA group (*r* = 0.214, *P* < 0.05). In patients with asthma, the prevalence of obesity was 64% in the presence of RA, and 40% in the absence of RA (*P* = 0.034).

**Conclusion:**

This study supports the positive association of RA with asthma, allergic rhinitis, and sinusitis. Our analysis suggests a notable interrelationship between the presence of asthma and higher BMI values in patients with RA, indicating that asthma is more obesity-related in patients with RA.

## Introduction

Rheumatoid arthritis (RA) is a chronic inflammatory disease characterized by polyarthritis that causes the joint deformity. RA develops as a consequence of complex interactions between genetic and environmental factors (1). Although various immune components can participate in pathogenesis, RA is generally believed to be a T cell-driven disease (2), especially involving pathogenic T helper 1 (Th1) and T helper 17 cells (3, 4). However, a recent study reported the expansion of T helper 2 (Th2) cells in the blood of treatment-resistant patients with RA, which suggested the contribution of Th2 cells to the RA pathogenesis (5).

Asthma, a Th2 cell disorder, is characterized by airway inflammation but can also propagate systemically (6). Accordingly, the relationship between asthma and systemic inflammatory disorders was previously elaborated (7–10). In addition to differences in immunologic mechanisms underlying RA and asthma, there are contrasting epidemiological patterns between the two conditions; RA tends to affect middle-aged to elderly females, while asthma is more prevalent in younger individuals (1, 11). Despite those differences, a large-scale prospective cohort study and meta-analysis revealed that asthma was an independent risk factor for RA (8). However, the association of RA with other asthma-related Th2 allergic diseases, including allergic rhinitis and atopic dermatitis, is controversial (12–16). Chronic obstructive pulmonary disease (COPD) and sinusitis, which could complicate asthma, were reported to be associated with RA (17–19). Nevertheless, their relationship with RA needs further confirmation. Briefly, the association of RA with bronchial asthma is considered to be robust, but not with asthma-related comorbidities.

Previously, the concept of an “allergic march” was suggested to describe the sequential development of allergic disorders (i.e., atopic dermatitis, asthma, and allergic rhinitis) (20). In addition, the term “asthma-related comorbidities” was used to refer to the co-occurrence of asthma-related immunologic, inflammatory, or metabolic diseases such as allergic rhinitis, sinusitis, and obesity (19). Immune and metabolic conditions are perturbed in RA (21), but the impact of RA on the relationship between asthma and its comorbidities has rarely been investigated.

This study aimed to reveal the association of RA with asthma and asthma-related comorbidities in a comprehensive manner. In addition, we investigated the comorbidity pattern of asthma as well as degree of interrelatedness between asthma and comorbidities in patients with and without RA. Herein, we conducted a population-based study using data from the Korean National Health and Nutrition Examination Survey (KNHANES).

## Materials and methods

### Study population and definition of variables

The KNHANES is a collection of nationally representative, cross-sectional, population-based health and nutritional surveys by the Korean Disease Control and Prevention Agency (KDCA) since 1998. KNAHNES includes a health interview, physical examination, laboratory tests, and nutritional questionnaire. The present study analyzed the 7 and 8th KNHANES (2016–2018, KNHANES-VII; 2019–2021, KNHANES-VIII) data from 2017 to 2019. Study participants had to meet all of the following inclusion criteria: (1) over 40 years old, (2) completed the questionnaires, and (3) completed spirometry tests. The maximum eligible age was 80 years. Medical data were obtained from the questionnaire or physical examination, which were performed by trained investigators following standardized procedures. Spirometry was performed for subjects >40 years of age using standardized equipment (model 1022; SensorMedics Corp, BD, Franklin Lakes, NJ, USA) according to guidelines of the American Thoracic Society/European Respiratory Society (22). Diagnosis of RA, asthma, allergic rhinitis, atopic dermatitis, sinusitis, and otitis media was defined based on the answers of self-reported questionnaire asking “Have you been diagnosed with the disease by a doctor?” (Yes/No) or “Do you take medicine or treatment for the disease?” Asthma patients were divided into two groups according to presence of airflow limitation [forced expiratory volume in 1 s (FEV_1_: L)/forced vital capacity (FVC: L) < 0.7] by spirometry test. COPD was defined as a spirometry result of airway obstruction (FEV_1_/FVC < 0.7) among adult >40 years of age without history of asthma according to the Global Initiative for Chronic Obstructive Lung Disease (GOLD) guidelines (23). Obesity was defined as a body mass index (BMI) greater than 25 kg/m^2^ based on the World Health Organization’s recommendations for the Asian population (24). The entire individual participated voluntarily and provided written informed consent. The KNHANES protocol was approved by the institutional review board of the KDCA. The data is available under following web site address.^1^

### Statistical analysis

For continuous variables, the subjects’ characteristics are presented as means and standard deviations, and for categorical variables, as relative frequencies. Continuous variables were compared using a *t*-test and categorical variables were compared using the chi-square test. For the multivariable logistic regression analysis of RA, two models were analyzed. Model 1 was adjusted for age, sex, body mass index (BMI), and current smoking status. It is widely recognized that individuals with allergic diseases, such as asthma, frequently suffer from other allergic and non-allergic conditions simultaneously. In order to determine the independent effect of comorbidities on RA, model 2 was further adjusted for the presence of asthma in addition to Model 1. Since cigarette smoking is known to be a risk factor for asthma, COPD, and RA, we also performed a sensitivity analysis to exclude the possible confounding effect of smoking by excluding subjects currently smoking (25). Additionally, to evaluate the probable interactions between independent variables on this association, an interaction term was added to Model 1. For the correlation matrix, correlation between variables were performed using the cor function in the stats package. For the correlation network, each variable is represented as a specific node. The size of the node reflects the prevalence, and the color of the node refers to the category of variables: demographics (yellow), correctable features (green), and comorbidities (coral). The links between nodes indicated statistically significant associations (*P* < 0.05). The thickness of the links correlated with the strength of their association (correlation coefficient), and color indicated the direction of association: blue for positive and pink for negative. The igraph package was used to visualize the correlation networks. To compare the strength of correlations between given variables in the RA and non-RA groups, we compared two correlations based on independent groups using Zou’s method (26), with an alpha level of 0.05 and a confidence level of 0.95. The cocor package was used to compare the correlation strength of the paired variables between patients with and without RA. To avoid bias, propensity score matching was performed between RA and non-RA groups with a 1:1 ratio based on age and sex using the MatchIt package (27, 28). Subsequently, a network analysis was conducted on the matched population. All statistical analyses were performed using R software (version 4.1.3).

## Results

### Characteristics of the participants

A total of 14,272 subjects aged >40 years who completed the questionnaires and spirometry tests from 2017 to 2019 were finally included. The patient inclusion process is depicted in Supplementary Figure 1. Among them, 334 (2.4%) had RA, 380 (2.8%) had asthma, 1,436 (11.0%) had allergic rhinitis, 693 (5.3%) had sinusitis, 545 (4.2%) had otitis media, 177 (1.4%) had atopic dermatitis, and 1,395 (12.5%) had COPD. The demographics and clinical characteristics of participants with and without RA are compared in Table 1. In the RA group, the mean age and proportion of women were higher, but the number of current smokers was lower. The prevalence of asthma was higher in the RA group (7.5% vs. 2.8%, *P* < 0.001), but that of other allergic diseases and COPD were not different between the two groups. The comorbidity patterns of asthma patients with and without RA are compared in Table 2. The prevalence of obesity was significantly higher in the asthma patients having concomitant RA (64.0% vs. 40.2%, *P* = 0.034). The prevalence of comorbidities is also illustrated with Venn diagrams comparing the groups with and without RA (Supplementary Figure 2).

**TABLE 1:** Characteristics of the participants with and without rheumatoid arthritis.

	Total (*N* = 14,272)	RA (–) (*N* = 13,384)	RA (+) (*N* = 334)	*P*-value
**Demographics**				
Age, mean ± SD, years	59.6 ± 11.9	59.5 ± 11.9	65.7 ± 11.0	**<0.001**
Female sex, *n* (%)	8,042 (56.3)	7,522 (56.2)	269 (80.5)	**<0.001**
BMI, mean ± SD, kg/m^2^	24.1 ± 3.3	24.1 ± 3.3	24.0 ± 3.4	0.429
Current smoking, *n* (%)	2,178 (15.9)	2,145 (16.0)	33 (9.9)	**0.003**
**Comorbidities, *n* (%)**				
Asthma	380 (2.9)	355 (2.8)	25 (7.5)	**<0.001**
With airflow limitation	120 (1.1)	115 (1.1)	5 (1.9)	0.331
COPD	1,395 (12.5)	1,368 (12.5)	27 (10.4)	0.351
Allergic rhinitis	1,436 (11.0)	1,393 (11.0)	43 (12.9)	0.319
Sinusitis	693 (5.3)	668 (5.3)	25 (7.5)	0.098
Otitis media	545 (4.2)	530 (4.2)	15 (4.5)	0.888
Atopic dermatitis	177 (1.4)	172 (1.4)	5 (1.5)	1.000
Obesity	4,926 (36.1)	4,805 (36.1)	120 (36.4)	0.970
Hypertension	4,580 (32.1)	4,412 (33.0)	168 (50.3)	**<0.001**
Hyperlipidemia	3,825 (29.8)	3,715 (29.6)	110 (36.3)	**0.014**
Diabetes mellitus	1,777 (13.0)	1,728 (12.9)	49 (14.7)	0.390

RA, rheumatoid arthritis; SD, standard deviation; BMI, body mass index; COPD, chronic obstructive pulmonary disease. *P*-values in bold are statistically significant.

**TABLE 2:** Characteristics of asthma patients with and without rheumatoid arthritis.

	Asthma (+)/RA (–) (*N* = 355)	Asthma (+)/RA (+) (*N* = 25)	*P*-value
**Demographics**			
Age, mean ± SD, years	64.4 ± 12.4	67.9 ± 8.9	0.163
Female sex, *n* (%)	224 (63.1)	22 (88.0)	**0.021**
BMI, mean ± SD, kg/m^2^	24.4 ± 3.8	26.5 ± 3.2	**0.009**
Obesity, *n* (%)	142 (40.2)	16 (64.0)	**0.034**
Current smoker, *n* (%)	41 (11.6)	2 (8.0)	0.830
**Comorbidities, *n* (%)**			
Allergic rhinitis	85 (23.9)	7 (28.0)	0.829
Sinusitis	38 (10.7)	6 (24.0)	0.092
Otitis media	26 (7.3)	1 (4.0)	0.824
Atopic dermatitis	14 (3.9)	1 (4.0)	>0.999
COPD	0 (0.0)	0 (0.0)	>0.999
**Pulmonary function**			
FEV_1_, mean ± SD, L	2.0 ± 0.7	1.9 ± 0.4	0.763
FEV_1_, mean ± SD,%	75.4 ± 18.7	86.3 ± 13.0	**0.022**
FVC, mean ± SD, L	2.8 ± 0.8	2.6 ± 0.5	0.264
FVC, mean ± SD,%	80.7 ± 14.6	87.0 ± 13.5	0.092
FEV_1_/FVC, mean ± SD	0.7 ± 0.1	0.7 ± 0.1	0.086

RA, rheumatoid arthritis; SD, standard deviation; BMI, body mass index; COPD, chronic obstructive pulmonary disease; FEV1, forced expiratory volume in 1 s; FVC, forced vital capacity. *P*-values in bold are statistically significant.

### Multivariable analysis for RA

**FIGURE 1:**
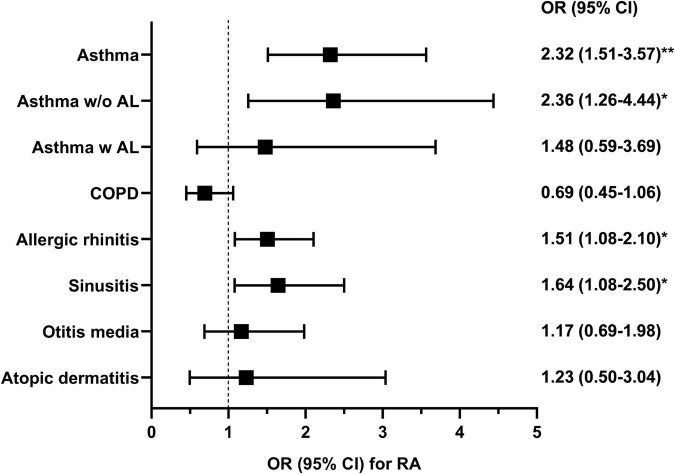
The odds ratio for rheumatoid arthritis in multivariable logistic regression analysis was adjusted by age, sex, current smoking status, and body mass index. The analysis was conducted using data from 13,718 patients with information about rheumatoid arthritis. Asthma w/o AL, asthma without airflow limitation; Asthma w AL, asthma with airflow limitation; COPD, chronic obstructive pulmonary disease; OR, odds ratio; CI, confidence interval; RA, rheumatoid arthritis. *P < 0.05 and **P < 0.001.

Since the distribution of age, sex, and current smoking status were different between the groups (Table 1), those confounding variables, in addition to BMI, were adjusted for multivariable logistic regression (Model 1). The detailed results of the multivariable analysis are summarized in Figure 1. RA was significantly associated with asthma [odds ratio (OR) 2.32; 95% confidence interval (CI) 1.51–3.57], but not with COPD (OR 0.69; 95% CI 0.45–1.06). The association between asthma and RA was relevant in asthma patients without airflow limitation but not in those with airflow limitation. RA was also associated with the presence of allergic rhinitis (OR 1.51; 95% CI 1.08–2.10) and sinusitis (OR 1.64; 95% CI 1.08–2.50). However, atopic dermatitis or otitis media was not significantly associated with RA. Considering the inter-correlation between allergic diseases, Model 1 was further adjusted for the presence of asthma (Model 2), and RA was still significantly associated with allergic rhinitis (OR 1.44; 95% CI 1.03–2.02) and sinusitis (OR 1.57; 95% CI 1.03–2.40), independent of asthma.

Sensitivity analysis was performed by excluding subjects currently smoking, and the results were quite similar (Supplementary Table 1). Furthermore, to analyze the effect of current smoking on this association, the interaction term between smoking and allergic diseases was added to Model 1. There was no significant interaction between current smoking status and above three diseases in association for RA (*P* for asthma = 0.76, *P* for allergic rhinitis = 0.66, and *P* for sinusitis = 0.44). To analyze the effect of sex on this association, the interaction term between sex and allergic diseases was added to Model 1; however, their interactions for RA were not significant (*P* for asthma = 0.56, *P* for allergic rhinitis = 0.83, and *P* for sinusitis = 0.48). The interaction between asthma and allergic rhinitis (*P* = 0.62) or sinusitis (*P* = 0.12) was not significant.

### Network analysis of the association between asthma and asthma-related comorbidities

To understand the correlation between demographics, asthma, asthma-related comorbidities, and RA, the correlation coefficients for these variables were calculated (Supplementary Table 2). Asthma was positively correlated with all disease entities in our analysis to varying degrees, except for COPD. The strongest correlations were observed between sinusitis and allergic rhinitis, sinusitis and otitis media, and asthma and allergic rhinitis.

**FIGURE 2:**
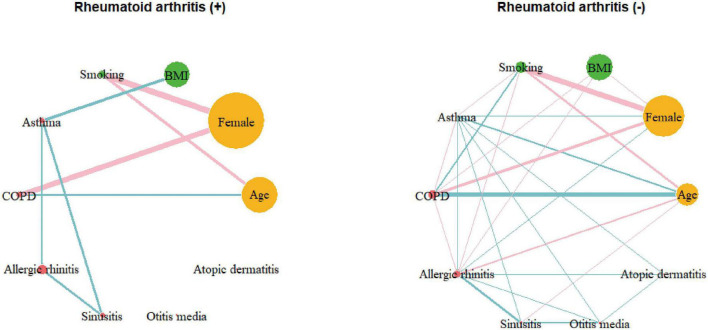
Correlation networks of the group with rheumatoid arthritis (*N* = 334) and those without rheumatoid arthritis (*N* = 13,384). Links between nodes indicate the existence of statistically significant association (*P* < 0.05). The thickness of links correlates with the strength of their association (correlation coefficient), and color means the direction of association: blue for positive and pink for negative. COPD, chronic obstructive pulmonary disease; BMI, body mass index.

To visualize these correlations more intuitively, a correlation network was constructed (Supplementary Figure 3). The network analysis exhibited a positive relationship between asthma and allergic rhinitis, sinusitis, otitis media, atopic dermatitis, BMI, and RA. To evaluate the difference in the pattern of the correlation network between the groups with and without RA, correlation networks were reconstructed separately for each group (Figure 2). The correlation coefficients for the groups with and without RA are summarized in Supplementary Tables 3, 4, respectively. In the RA network, a positive correlation was found between asthma and BMI, whereas the correlation network of the group without RA showed no significant relationship between asthma and BMI. In the RA group, asthma was associated with comorbidities, such as allergic rhinitis and sinusitis, but not with otitis media or atopic dermatitis. Interrelations between asthma, sinusitis and allergic rhinitis were observed in both groups, however, the strength of the correlation between asthma and sinusitis was significantly stronger in patients with RA (Supplementary Table 5). To avoid bias driven by demographic differences, we matched the two groups by age and sex (Supplementary Table 6). We also obtained separate correlation coefficients and networks for the two groups after matching. Similar to the analysis of the total population, significant associations of asthma with BMI and sinusitis were found only in the RA group (Supplementary Figure 4).

## Discussion

We found that the prevalence of asthma was higher in RA patients than in non-RA patients (7.5% vs. 2.8%, *P* < 0.001). After multivariable adjustment, asthma, allergic rhinitis, and sinusitis were positively associated with RA. Among them, asthma had the strongest association with RA (OR 2.32; 95% CI 1.51–3.57). These associations were consistently found in logistic regression analyses using interaction terms or sensitivity analysis, supporting the unbiased nature of the results. The comorbidity pattern was different in asthma patients according to the presence of RA; obesity was more common in patients with asthma and concomitant RA. According to network analysis, the interconnection between asthma and sinusitis was stronger in patients with RA. Moreover, asthma and BMI were positively correlated only in RA patients. Our data suggest that asthma in RA patients is more obesity-related, which could be particularly meaningful since treatment refractoriness of asthma is partly attributable to obesity (29).

Among allergic diseases, asthma has been proven to be associated with RA (10). Longitudinal analysis revealed a higher likelihood of developing RA in patients with asthma (17). Conversely, a higher rate of asthma development has been observed in patients with RA (9). Lung inflammation was noted as one of the major pathogenic mechanisms involved in the development of RA. Mucosal inflammation of the respiratory system upregulates citrullination, which can provoke the formation of anti-citrullinated antibodies, the major contributors to RA pathogenesis (30, 31). Asthma, which is caused by Th2 cell-rich airway inflammation, is also known to enhance citrullination reactions, and a higher positive rate of anti-citrullinated antibodies has been demonstrated in the peripheral blood of asthma patients. Consistently, the presence of asthma elevated the risk of seropositive RA but not the risk of overall RA (32). Another finding of our study was that their association was only relevant in the group without airflow limitation. There is a possibility that the group of patients with asthma with airflow limitation might have included some patients with COPD. As asthma and COPD commonly display overlapping features (33), it is often difficult to differentiate them without bronchodilator response tests or bronchoprovocation tests (34). Nevertheless, the proportion of patients with airflow limitation in asthma that we reported was comparable to a previous report [38% (120/380) vs. 31% (74/239), *P* = 0.87] (35). The overall prevalence of asthma and COPD in our data was also consistent with Korean epidemiologic data (36, 37). The exact mechanism is unclear for why RA is not associated with asthma with airflow limitation but could be attributable to the heterogeneity of this population.

The association between RA and COPD has remained controversial. Some studies reported positive associations between these diseases, independent of the smoking effect (17, 38). An increase in citrullinated proteins was detected in the lungs of patients with COPD as well (39). Contrarily, other studies have shown insignificant relationships across different stages of lung function (40, 41). No relationship between COPD and RA was observed in the present study. This could be due to the heterogeneous definitions of COPD in each study and the variance in adjustment for the smoking effect.

Positive association between RA and allergic rhinitis had been reported by some studies (15). Our data showed that the association with allergic rhinitis was weaker than that with asthma, which is consistent with the results of previous publications (42). This is probably attributable to the absence of mucosal inflammation in the lower respiratory tract in allergic rhinitis, which is the major location for citrullination. In the present study, sinusitis was found to be positively associated with RA. A recent study reported positive association of sinusitis and pharyngitis with RA, implicating the contribution of upper airway mucosal inflammation to RA pathogenesis (18). Previous publications showed a lower prevalence of atopic dermatitis in RA patients than in healthy controls. This was attributed to the Th1-dominant autoimmunity of RA, which may counteract the Th2 immunity of atopic dermatitis (43). A representative study suggesting a higher incidence of RA in patients with atopic dermatitis performed an analysis of participants aged 40 years or younger, most of whom were children or adolescents (16). Our analysis, however, included participants 40 years or older. Since a recent report highlighted the different characteristics between pediatric and adult atopic dermatitis, the findings from the younger and the older participants could be different (44).

One of the striking findings of our study is the association of higher BMI with asthma in patients with RA. Among the heterogenous phenotypes of asthma, obesity-related asthma is a subgroup that is difficult to manage. Due to the increased production of inflammatory cytokines from adipose tissue (45), obesity-related asthma tends to have a Th1-skewed response to inflammatory stimuli and non-atopic features, such as a low fraction of exhaled nitric oxide (46). Our results indicated that there was no significant correlation between asthma and atopic dermatitis in patients with RA. On the other hand, a more pronounced relationship was discovered between asthma and sinusitis in those with RA. Taking into account that atopic dermatitis is mediated by Th2 immunity and that Th1 immunity plays dominant role in the subtype of sinusitis (47), these overall findings suggest that asthma in RA might be more Th1-skewed. Unfortunately, our analysis lacked immune profiling of the subjects. Therefore, further basic or translational studies are needed to confirm the association.

Significant relationships between asthma, allergic rhinitis, and sinusitis were observed in both groups with and without RA. The concept, “allergic march,” suggests a strong relationship between allergic diseases, and was also supported by several reports (20). Sinusitis often develops as a complication of allergic rhinitis, and the co-occurrence of asthma and sinusitis is frequently observed (48). Thus, these interrelations are consistent with traditional concepts. However, the strengthened relationship between asthma and sinusitis in patients with RA is a novel finding of the present study.

The limitations of this study include followings. First, this was a population-based cross-sectional study that did not include detailed information on disease activity, the diagnostic criteria applied, serologic status, medication history, other autoimmune diseases, other environmental risk factors, or the number of cigarettes smoked. In addition, laboratory findings such as blood eosinophil count, C-reactive protein level, and bronchodilator response on spirometry or methacholine bronchoprovocation test for asthma severity were not measured. This is attributable to the nature of the secondary data we used. Second, atopic dermatitis was infrequent because we included participants aged >40 years. The design for this study population was intended to adjust for age-related confounding effects, but atopic dermatitis is less frequent in individuals over 40 (49). Thus, the relationship between RA and atopic dermatitis may have been underestimated. Lastly, causal relationships cannot be found in the significant associations because of the cross-sectional design of the study and need to be confirmed with further longitudinal studies.

In conclusion, RA was significantly positively associated with asthma (OR 2.32; 95% CI 1.51–3.57), allergic rhinitis (OR 1.51; 95% CI 1.08–2.10), and sinusitis (OR 1.64; 95% CI 1.08–2.50), but not with COPD, atopic dermatitis, or otitis media. The network analysis exhibited interrelationships between asthma and sinusitis was more accentuated in the RA group compared to the non-RA group. Most strikingly, network analysis revealed a distinct positive link between asthma and higher BMI values in patients with RA.

## Data availability statement

Publicly available datasets were analyzed in this study. This data can be found here: https://www.data.go.kr/data/15076556/fileData.do.

## Ethics statement

The KNHANES protocols were reviewed and approved by the Research Ethics Review Board of National Center for Health Statistics. The patients/participants provided their written informed consent to participate in this study.

## Author contributions

H-KK had full access to all of the data in the study and takes responsibility for the integrity of the data and the accuracy of the data analysis. H-KK and JGK contributed to the study concept and design and drafted the manuscript and contributed to the statistical analysis. H-KK, JGK, J-HL, and JK contributed to the acquisition, analysis, or interpretation of the data, and contributed to the critical revision of the manuscript for important intellectual content. All authors read and approved the final manuscript.

## Footnotes

1
https://www.data.go.kr/data/15076556/fileData.do
