# Supplementary material for: Association of rheumatoid arthritis with bronchial asthma and asthma-related comorbidities: A population-based national surveillance study
Source: Front Med (Lausanne). 2023 Mar 10;10:1006290. doi: 10.3389/fmed.2023.1006290 (PMC10036351; doi:10.3389/fmed.2023.1006290)
Supplement: Supplementary file 1 [file Data_Sheet_1.docx]

**SUPPLEMENTARY MATERIAL**

**This file includes:**

**Supplementary Figure 1**

**Supplementary Figure 2**

**Supplementary Figure 3**

**Supplementary Figure 4**

**Supplementary Table 1**

**Supplementary Table 2**

**Supplementary Table 3**

**Supplementary Table 4**

**Supplementary Table 5**

**Supplementary Table 6**

KNHANES (2017 to 2019)

(n=24,229)

Subjects who completed questionnaires

(n=23,047)

Subjects aged 40 years or older

and completed spirometry

(n=14,272)

**Supplementary Figure 1.** Flow chart of patient selection

KNHANES, the Korean National Health and Nutrition Examination Survey.


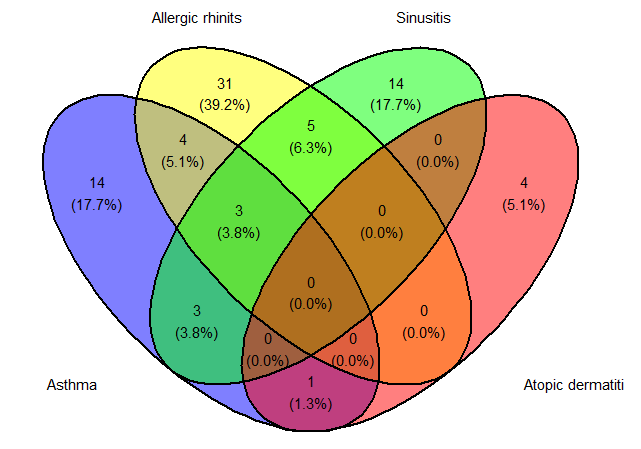

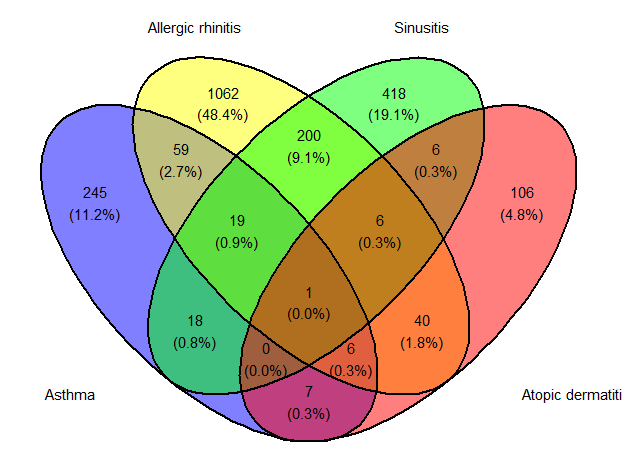


Rheumatoid arthritis (-)

Rheumatoid arthritis (+)

**Supplementary Figure 2**. Venn Diagrams displaying prevalence and overlapping patterns of asthma and asthma-related comorbidities


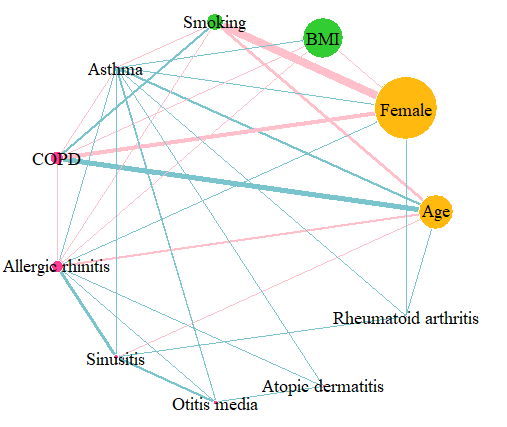


**Supplementary Figure 3**. Correlation network. Links between nodes indicate an existence of statistically significant association (*P* < 0.05). The thickness of links correlates with strength of their association (correlation coefficient), and color means direction of association: blue for positive and pink for negative

BMI, body mass index; COPD, chronic obstructive pulmonary disease.

**Supplementary Figure 4.** Correlation networks derived from the RA group (*N* = 334) and the non-RA group matched based on age and sex (*N* = 334). Links between nodes indicate an existence of statistically significant association (*P* < 0.05). The thickness of links correlates with strength of their association (correlation coefficient), and color means direction of association: blue for positive and pink for negative

BMI, body mass index; COPD, chronic obstructive pulmonary disease.

**Supplementary Table 1**. Sensitivity analysis excluding subjects currently smoking

|  |  | **Rheumatoid arthritis** | |
| --- | --- | --- | --- |
|  |  | OR^*^ | 95% CI |
| **Asthma** |  | 2.368 | 1.512-3.708 |
|  | Without airflow limitation | 2.531 | 1.342-4.774 |
|  | With airflow limitation | 1.705 | 0.678-4.282 |
| **COPD** |  | 0.730 | 0.455-1.172 |
| **Allergic rhinitis** |  | 1.572 | 1.111-2.226 |
| **Sinusitis** |  | 1.564 | 0.995-2.446 |
| **Otitis media** |  | 1.316 | 0.772-2.241 |
| **Atopic dermatitis** |  | 1.155 | 0.421-3.172 |

OR, odds ratio; CI, confidence interval; COPD, chronic obstructive pulmonary disease.

*Adjusted by age, sex, and body mass index.

**Supplementary Table 2**. Correlation matrix between demographic characteristics and comorbidities

|  | Age | Female | BMI | Asthma | COPD | AR | Sinusitis | OM | AD | RA |
| --- | --- | --- | --- | --- | --- | --- | --- | --- | --- | --- |
| Age | 1 | -0.005 | 0.011 | 0.097^*^ | 0.270^*^ | -0.126^*^ | -0.034^*^ | -0.003 | -0.018 | 0.080^*^ |
| Female |  | 1 | -0.084^*^ | 0.030^*^ | -0.209^*^ | 0.067^*^ | 0.001 | 0.010 | -0.004 | 0.075^*^ |
| BMI |  |  | 1 | 0.021^*^ | -0.068^*^ | -0.021^*^ | 0.005 | 0.009 | -0.007 | -0.004 |
| Asthma |  |  |  | 1 | -0.070^*^ | 0.077^*^ | 0.053^*^ | 0.027^*^ | 0.042^*^ | 0.052^*^ |
| COPD |  |  |  |  | 1 | -0.042^*^ | -0.002 | -0.003 | -0.011 | -0.011 |
| AR |  |  |  |  |  | 1 | 0.175^*^ | 0.065^*^ | 0.077^*^ | 0.014 |
| Sinusitis |  |  |  |  |  |  | 1 | 0.098^*^ | 0.003 | 0.026^*^ |
| OM |  |  |  |  |  |  |  | 1 | 0.048^*^ | -0.004 |
| AD |  |  |  |  |  |  |  |  | 1 | 0.002 |
| RA |  |  |  |  |  |  |  |  |  | 1 |

BMI, body mass index; COPD, chronic obstructive pulmonary disease; AR, allergic rhinitis; OM, otitis media; AD, atopic dermatitis, RA, rheumatoid arthritis.

**P* < 0.05.

**Supplementary Table 3**. Correlation matrix of groups with rheumatoid arthritis

|  | Age | Female | BMI | Smoking | Asthma | COPD | AR | Sinusitis | OM | AD |
| --- | --- | --- | --- | --- | --- | --- | --- | --- | --- | --- |
| Age | 1.000 |  |  | -0.212 |  | 0.153 | -0.127 |  |  |  |
| Female |  | 1.000 |  | -0.398 |  | -0.394 |  |  |  |  |
| BMI |  |  | 1.000 |  | 0.214 |  |  |  |  |  |
| Smoking |  |  |  | 1.000 |  |  |  |  |  |  |
| Asthma |  |  |  |  | 1.000 |  | 0.127 | 0.178 |  |  |
| COPD |  |  |  |  |  | 1.000 |  |  |  |  |
| AR |  |  |  |  |  |  | 1.000 | 0.161 |  |  |
| Sinusitis |  |  |  |  |  |  |  | 1.000 |  |  |
| OM |  |  |  |  |  |  |  |  | 1.000 |  |
| AD |  |  |  |  |  |  |  |  |  | 1.000 |

Only statistically significant correlations are presented.

BMI, body mass index; AR, allergic rhinitis; OM, otitis media; AD, atopic dermatitis.

**Supplementary Table 4**. Correlation matrix of groups without rheumatoid arthritis

|  | Age | Female | BMI | Smoking | Asthma | COPD | AR | Sinusitis | OM | AD |
| --- | --- | --- | --- | --- | --- | --- | --- | --- | --- | --- |
| Age | 1.000 |  |  | -0.144 | 0.093 | 0.274 | -0.129 | -0.037 |  |  |
| Female |  | 1.000 | -0.084 | -0.369 | 0.025 | -0.207 | 0.066 |  |  |  |
| BMI |  |  | 1.000 |  |  | -0.068 | -0.022 |  |  |  |
| Smoking |  |  |  | 1.000 | -0.023 | 0.126 | -0.050 |  |  |  |
| Asthma |  |  |  |  | 1.000 | -0.069 | 0.074 | 0.046 | 0.028 | 0.041 |
| COPD |  |  |  |  |  | 1.000 | -0.042 |  |  |  |
| AR |  |  |  |  |  |  | 1.000 | 0.175 | 0.069 | 0.080 |
| Sinusitis |  |  |  |  |  |  |  | 1.000 | 0.099 |  |
| OM |  |  |  |  |  |  |  |  | 1.000 | 0.049 |
| AD |  |  |  |  |  |  |  |  |  | 1.000 |

Only statistically significant correlations are presented.

BMI, body mass index; AR, allergic rhinitis; OM, otitis media; AD, atopic dermatitis.

**Supplementary Table 5**. The comparison of two Pearson’s correlation coefficients between the group without RA and with RA

| **Variables** | **95% CI of the difference between the two correlations^†^** |
| --- | --- |
| Asthma – allergic rhinitis | -0.1619 to 0.0542 |
| Asthma – sinusitis | -0.2354 to -0.0247^*^ |
| Allergic rhinitis - sinusitis | -0.0901 to 0.1216 |
| Asthma – body mass index | -0.2688 to -0.1335^*^ |

CI, confidence interval.

*indicates statistically significant difference. 95% CI below zero means the stronger positive correlation between the given variables in the RA group.

†Null hypothesis is rejected when interval does not include 0.

**Supplementary Table 6**. Characteristics of participants with rheumatoid arthritis and matched control

|  | **RA (-)**  (N=334) | **RA (+)**  (N=334) | *P-value* |
| --- | --- | --- | --- |
| **Demographics** |  |  |  |
| Age, mean ± SD, years | 65.7 ± 11.0 | 65.7 ± 11.0 | >0.999 |
| Female sex, n (%) | 269 (80.5) | 269 (80.5) | >0.999 |
| BMI, mean ± SD, kg/m^2^ | 24.0 ± 3.3 | 24.0 ± 3.4 | 0.991 |
| Current smoking, n (%) | 22 (6.6) | 33 (9.9) | 0.166 |
| **Comorbidities, n (%)** |  |  |  |
| Asthma | 11 (3.3) | 25 (7.5) | **0.026** |
| With airflow limitation | 5 (1.5) | 5 (1.9) | >0.999 |
| COPD | 27 (10.4) | 27 (10.4) | 0.901 |
| Allergic rhinitis | 33 (9.9) | 43 (12.9) | 0.273 |
| Sinusitis | 22 (6.6) | 25 (7.5) | 0.762 |
| Otitis media | 20 (6.0) | 15 (4.5) | 0.487 |
| Atopic dermatitis | 3 (0.9) | 5 (1.5) | 0.722 |
| Obesity | 15 (35.2) | 120 (36.4) | 0.812 |
| Hypertension | 165 (50.2) | 187 (56.2) | 0.142 |
| Hyperlipidemia | 117 (35.0) | 129 (38.5) | 0.378 |
| Diabetes mellitus | 47 (14.1) | 48 (14.4) | >0.999 |

RA, rheumatoid arthritis; SD, standard deviation; BMI, body mass index; COPD, chronic obstructive pulmonary disease.

*P* values in bold are statistically significant.
